# Toward Water‐Immersion Programmable Meta‐Display

**DOI:** 10.1002/advs.202205581

**Published:** 2022-12-18

**Authors:** Shuai Wan, Chenjie Dai, Zhe Li, Liangui Deng, Yangyang Shi, Wanlin Hu, Guoxing Zheng, Shuang Zhang, Zhongyang Li

**Affiliations:** ^1^ Electronic Information School Wuhan University Wuhan 430072 P. R. China; ^2^ Wuhan Institute of Quantum Technology Wuhan 430206 P. R. China; ^3^ Department of Physics The University of Hong Kong Pokfulam Road Hong Kong 999077 P. R. China; ^4^ School of Microelectronics Wuhan University Wuhan 430072 P. R. China; ^5^ Suzhou Institute of Wuhan University Suzhou 215123 P. R. China

**Keywords:** independent‐programmable, meta‐holography, meta‐nanoprinting, tunable meta‐display, water‐immersion

## Abstract

Heading toward next‐generation intelligent display, dynamic control capability for meta‐devices is critical for real world applications. Beyond the conventional electrical/optical/mechanical/thermal tuning methods, liquid immersion recently has emerged as a facile tuning mechanism which is easily accessible (especially water) and practically implementable for large tuning area. However, due to the longstanding and critical drawback of lacking independent‐encoding capability, the state‐of‐art immersion approach remains incapable of pixel‐level programmable switching. Here a water‐immersion tuning scheme with pixel‐scale programmability for dynamic meta‐displays is proposed. Tunable meta‐pixels can be engineered to construct spectral selective patterns at prior‐/post‐ immersion states, such that a metasurface enables pixel‐level transforming animations for dynamic multifield meta‐displays, including near‐field dual‐nanoprints and far‐field dual‐holographic displays. The proposed water‐immersion programmable approach for meta‐display, benefitting from its large tuning area, facile operation and strong repeatability, may find a revolutionary path toward next‐generation intelligent display with practical applications in dynamic display/encryption, information anticounterfeit/storage, and optical sensors.

## Graphical abstract

The proposed water‐immersion programmable meta‐device, for the first time, intriguingly achieves pixel‐programmable water‐immersion switch for multifield meta‐display transformation through screening tunable meta‐pixels to construct spectral programmability, beyond simply coloring alteration, or on‐and‐off switch. The practical water‐immersion mechanism, which enjoys large tuning area, great simplicity in operation and strong repeatability, may find a revolutionary path toward the next‐generation intelligent display technology.

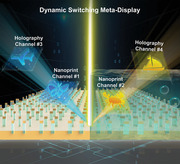

## 1 Introduction

Meta‐optics, as a burgeoning branch in the field of optics, creates unprecedented possibilities to manipulate light at will. Among all of its various promising functionalities and applications, including perfect optical absorber,^[^ ^1^, ^2^, ^3^ ^]^ meta‐lens,^[^ ^4^, ^5^, ^6^, ^7^ ^]^ arbitrary beam steering/merging,^[^ ^8^, ^9^, ^10^, ^11^ ^]^ the invention of meta‐display triggers a prominent revolution for image exhibition and projection with tremendous improvement in image quality, resolution, and information storage capability compared to conventional means. On one hand, for the near‐field exhibition of nanoprints,^[^ ^12^, ^13^, ^14^, ^15^ ^]^ an engineered meta‐pixel with nanoantennas/nanoparticles can provide superior structural coloring performance with ultra‐high resolution (>100 000 dpi) and ultralarge gamut (>180% of sRGB gamut).^[^ ^11^ ^]^ On the other hand, the far‐field display of meta‐holography^[^ ^16^, ^17^, ^18^, ^19^, ^20^, ^21^, ^22^, ^23^, ^24^ ^]^ conquers the limitations of traditional diffractive optical elements (DOE) and achieves powerful multiplexing capability with multi‐channels (>2–8),^[^ ^18^, ^19^, ^20^ ^]^ high‐efficiency (>90%),^[^ ^17^ ^]^ and large viewing‐angle (>120°).^[^ ^24^ ^]^ With such unique potential and excellent performance, meta‐display makes a critical leap toward advanced display applications, including virtual/augmented reality (VR/AR),^[^ ^19^ ^]^ information storage/encryption,^[^ ^20^, ^21^ ^]^ and anticounterfeiting displays.^[^ ^14^ ^]^

Heading toward the next‐generation intelligent display, the practical tuning capability for meta‐device is critical for its wide application in real‐life scenarios. Various conventional tuning schemes, including electrical,^[^ ^25^, ^26^, ^27^, ^28^, ^29^, ^30^, ^31^ ^]^ thermal,^[^ ^5^, ^14^ ^]^ chemical,^[^ ^32^, ^33^ ^]^ mechanical,^[^ ^34^, ^35^ ^]^ and optical^[^ ^36^, ^37^, ^38^ ^]^ stimuli, have been extensively studied for enabling dynamic control of meta‐display. Apart from the above investigated methods, liquid immersion tuning,^[^ ^39^, ^40^, ^41^, ^42^, ^43^, ^44^, ^45^, ^46^ ^]^ emerging as a facile and practical scheme, has been tentatively explored. Compared with the sophisticated and demanding requirement of optical/electrical tuning, liquid immersion tuning is more commonly accessible and implementable, in addition to the benefits of large tuning area and good repeatability.

However, one of the most critical drawbacks of the state‐of‐the‐art environmental immersion tuning is the lack of programmable capability to enable pixel‐level switching between multi‐fold meta‐displays. Because most of the liquid/water‐immersion tuning mechanism relies on altering the environmental refractive index surrounding the meta‐pixel patterns, the previously demonstrated meta‐display tuning usually exhibits a straightforward structural color alternation or simply an on‐and‐off switching behavior based on the overall pattern's collective spectral modulation. Therefore, it is particularly challenging yet highly desirable for such facile immersion tuning method to create independent encoding freedom and to enable the pixel‐scale programmable multi‐fold meta‐display between the prior‐ and post‐ immersion states.

Here, we propose and realize a facile water‐immersion tuning scheme to realize programmable switching between multi‐fold meta‐displays with independent encoding freedom. Through architectural screening and by exploiting the drastic spectral alternation of the nanostructured resonators when immersed with water, tunable meta‐pixel basis is elaborately selected to provide independent spectral amplitude for prior‐ and post‐ immersion states. To fully break the entire collective tuning performance and to achieve pixel‐level programmability, an amplitude transformation matrix from the designed metasurface pattern at dry/wet state is generated through optimization based on a modified simulated annealing algorithm (SAA). The designed metasurface is capable of simultaneously exhibiting multi‐fold meta‐displays, that is, near‐field nanoprints and far‐field holographic images. Through water‐immersion tuning of the prior‐/post‐ moisture states at the pixel level, a single metasurface acquires completely independent encryption freedom for both near‐/far‐field meta‐image transformations.

## 2 Results and Discussion

**Figure 1:**
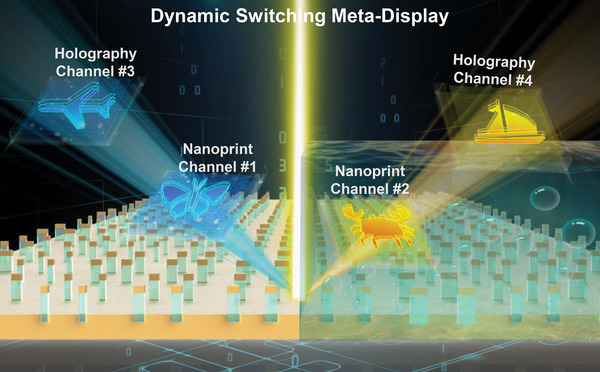
Conceptual schematic for the water‐immersion programmable dynamic switch of multi‐fold meta‐display. Quad‐fold imaging channels are enabled from a single metasurface at the prior‐ and post‐ immersion states, such as independent‐encoded near‐field nanoprint images of a butterfly and a crab and far‐field holographic images of an airplane and a ship.

**Figure** **1** schematically illustrates the basic concept for our proposed water‐immersion programmable dynamic multi‐field meta‐display. For the near‐field nanoprints, the designed metasurface would switch at the pixel‐level between the image of a butterfly in air or that of a crab in water. In the meanwhile, it can also be actively tuned at the hologram pixel‐scale to project the holographic image of an airplane in the far‐field in the dry state or that of a ship in the immersed state.

**Figure 2:**
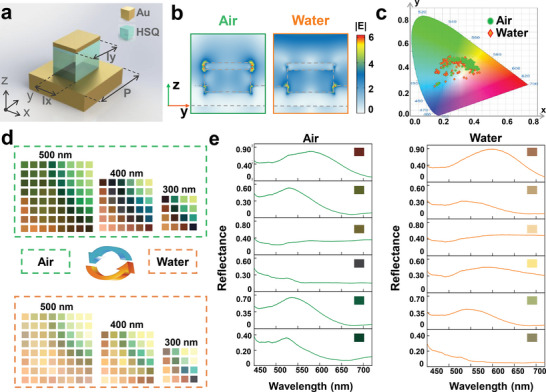
The optical response of meta‐pixels. a) Structural diagram of the meta‐pixels. b) Electric‐field intensity contours of the meta‐pixel at *y‐z* plane in different surrounding refractive indexes from air (*n* = 1) to water (*n* = 1.33) under the incident light with *y‐*polarization at 590 nm. The size parameter of the meta‐pixel is *P* = 500 nm, *l_x_* = 200 nm, and *l_y_* = 300 nm. c) The CIE 1931 plot for the experimentally calculated structural color palettes of 116 meta‐pixels at prior‐ and post‐ immersion states. d) Experimentally recorded color palettes of 116 meta‐pixels with different periods and geometrical parameters at prior‐ and post‐ immersion states, respectively. e) Experimental reflection spectra of selected six meta‐pixels in prior‐ and post‐ immersion conditions. The insets are their displayed colors under the bright‐field microscope.

To achieve the water‐immersion switchable meta‐display, a large number of architectural meta‐pixels are designed to exhibit particular spectral responses under air/water immersion, to serve as potential building blocks. As schematically depicted in **Figure** **2**a, each meta‐pixel is designed as a nanopillar‐cavity of hydrogen silsesquioxane (HSQ) (120 nm thick) covered by an Au thin film (30 nm thick) atop a thick metallic mirror (70 nm thick). More detailed explanation of architectural processing is in the Experimental Section. To unveil the underlying mechanism for inducing the spectral variation with water‐immersion, we calculate and compare the corresponding electric‐field intensity distributions at *y‐z* cross‐section planes for a representative meta‐pixel architecture (*P* = 500 nm, *l_x_* = 200 nm, *l_y_* = 300 nm) under air and water immersion conditions. As shown in Figure 2b, the excited plasmonic resonant mode in the air is noticeably distinct from that in water immersion. Specifically, the major resonance hotspots are shifted from the top edges to the bottom edges of the nanocavity when immersed in water (refractive index 1.33), thus facilitating decoupled spectral responses for dry/water immersion conditions.

To illustrate the capability of the selected meta‐pixels to independently manipulate the spectral response during the water‐immersion tuning, we compare the color variation for representative experimental spectra with prior‐/post‐ immersion, as shown in Figure 2c–e. By retrieving the dry/immersed structural colors and plotting them into the same CIE 1931 colormap (Figure 2c), it is observed that the gamut area is slightly shifted and increased after water‐immersion operation. The experimentally measured colors from 50 µm size square patterns also become noticeably shifted and brighter than those in air (Figure 2d). This immersion‐induced spectral variation is further illustrated by experimentally recorded reflection spectra of six selected meta‐pixels in air and water conditions, as shown in Figure 2e. More demonstrations of multiple pattern sizes (2–50 µm) can be found in Section S1 (Supporting Information). Note that the use of 2 µm size square meta‐pixel would boost the meta‐display resolution limit above 12 700 dpi, which is promising for advanced display applications.

**Figure 3:**
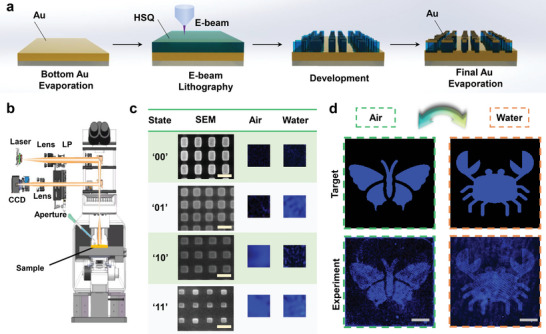
Water‐immersion pixel‐programmable meta‐display to exhibit dynamic nanoprint image switch via water‐immersion tuning. a) Schematic illustration of the fabrication process for pixel‐programmable metasurface pattern without lift‐off requirement. b) Experimental setup to characterize the water‐immersion switchable nanoprint image based on the angle‐resolved microscope spectrometer (ARMS, Ideaoptics Inc). c) Scanning electron microscopic images (SEM) and the optical microscopic images at prior‐ and post‐ immersion conditions under 480 nm laser light. The selected four meta‐pixels can provide independent‐encoding reflection amplitude. The scale bars in SEM images are 500 nm. d) Target and experimental near‐field nanoprint images with reasonable image enhancement at prior‐ and post‐ immersion states. The nanoprint image could actively transform from a butterfly in the air state to a crab in the water‐immersion state. The scale bars correspond to 100 µm.

The next critical step for dynamic image switching is to establish a meta‐pixel matrix transformation for the water‐immersion process. Based on the selected meta‐pixels from Figure 2 that can be tuned to exhibit different spectral responses, as a proof‐of‐concept, we first design and demonstrate dynamically switchable images of near‐field nanoprints with water‐immersion method. The meta‐display samples are fabricated by thermal evaporation deposition for metallic layers, HSQ spin‐coating, and patterning via electron beam lithography (EBL) (**Figure** **3**a). For the observation of dynamic switching between different near‐field displays, we utilize a microscopic optical system with a 10× objective lens (NA = 0.25) and a colored charge‐coupled device (CCD), as shown in Figure 3b.

Here, we encode four meta‐pixels into binary states as “00,” “01,” “10,” and “11” (Figure 3c), where “0” and “1” represent the low/high states of the reflection amplitude (*A*) contrast at the operation wavelength; the first and second digit present the prior‐ and post‐ immersion states, respectively. Specifically, the reflection amplitudes of the above four meta‐pixels are given by “*A*_air_ = *A*_water_ = 0,” “*A*_air_ = 1 and *A*_water_ = 0,” “*A*_air_ = 0 and *A*_water_ = 1,” and “*A*_air_ = *A*_water_ = 1,” respectively.

Through screening large numbers of fabricated meta‐pixels, we successfully retrieve and select the desired four encoded states that can serve as the amplitude‐programmable basis for the water‐immersion tuning. The scanning electron microscopic (SEM) images and the corresponding brightness under laser illumination at the wavelength of 480 nm are shown in Figure 3c, which match well with the designed binary states. The fabricated sample has an area of 500 × 500 µm^2^ with a meta‐pixel resolution of ≈12 700 dpi. Figure 3d shows the experimentally captured near‐field image that dynamically transforms from the image of a butterfly in air into that of a crab when immersed in water. Despite the stray light noise, uneven light source spot, and nanofabrication defects, the two completely independent‐encoded images match reasonably well with the target images. The dynamic process of the image transformation in real‐time can be found in Movie S1 (Supporting Information). The entire switching period of the dynamic image transformation can be ≈1.5 s. More detailed information regarding the modulation rate can be found in Section S2 (Supporting Information).

**Figure 4:**
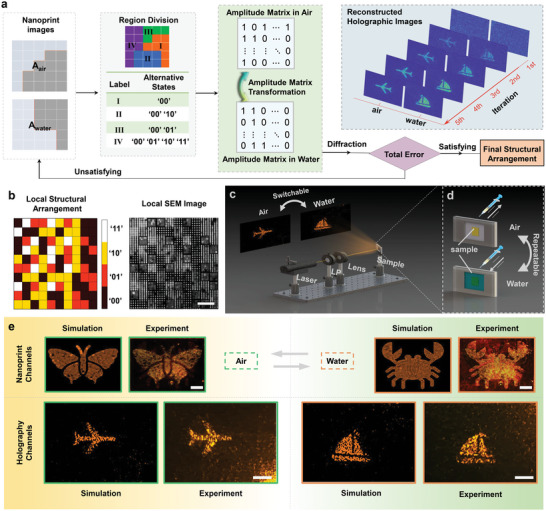
Optimization flow chart and experimental characterization of the switchable multi‐field meta‐display via water‐immersion tuning. a) Flow chart of the iterative calculation process for optimizing the water‐immersion programmable quad‐fold meta‐display by the modified simulated annealing algorithm (SAA). b) Local diagram of the optimized structural arrangement and the corresponding SEM image of the fabricated sample. The scale bar is 4 µm. c) Experimental setup to characterize the water‐immersion switchable meta‐holographic images. d) Schematic for water‐immersion capsule for tuning the immersion‐state. e) Simulated and measured water‐immersion independent‐encoded multi‐field meta‐images. The meta‐display is switched between the independent‐encoded dual‐nanoprint and dual‐holographic images by actively immersion tuning between air and water states. The reconstructed holographic images exhibit the respective designated area and are reasonably processed with brightness/contrast enhancement. The scale bars in nanoprint and holographic images correspond to 100 µm and 1 cm, respectively.

To fully unfold the switching capability of the multi‐field meta‐display for increasing the multiplexing channels, another metasurface is designed to simultaneously exhibit both near‐field and far‐field meta‐displays by optimization through the modified SAA. **Figure** **4**a illustrates the iterative calculation and optimization process to reconstruct high‐quality far‐field holographic channels through balancing between the near‐field amplitude uniformity and far‐field image quality. The iterative optimization process begins with dividing the pattern into four regions from I to IV (“00,” “01,” “10,” and “11”), according to the target near‐field images and the immersion conditions. For each region, multiple alternative states are available for selection (instead of only a single previous state) to program the multi‐field display when carefully balancing the near‐field amplitude arrangement. For instance, both state options of “00” and “01” can be encoded for region‐II which should collectively exhibit low brightness in air and high brightness in water; Likewise, the state options of “00,” “01,” “10,” and “11” can be mixed and utilized for the region‐IV which represent high brightness in both air and water. Despite the sacrifice of the near‐field amplitude uniformity, it is not anticipated to strongly impact the overall image quality. The amplitude matrices in air/water can be accordingly derived from the structural arrangement and the environmental transformation. The far‐field holographic images prior‐/post‐ immersion are eventually reconstructed via Fast Fourier transformation (FFT). Through comparing and evaluating the deviation of the reconstructed holographic images from the ideal target, the temporary optimal solution is either kept as the previous one or replaced by the current solution. Typically, through 1st to 5th iterations, the optimal solution rapidly converges (Figure 4a). More detailed information regarding the image quality analysis and improvement potential can be found in Section S3 (Supporting Information).

Figure 4b shows the local diagram of the optimized meta‐pixel arrangement with the corresponding SEM image of the fabricated sample. The target near‐field displays contain a pixel matrix size of 210 × 300, and the fabricated sample area is 420 × 600 µm^2^, where each pixel has an area of 2 × 2 µm^2^ to reduce the adjacent meta‐pixel coupling.

Figure 4c illustrates the optical measurement setup for capturing the reconstructed holographic images and the near‐field displays. For the in‐ situ water‐immersion characterization, we customize a liquid‐immersion capsule to dynamically control the sample immersion state and the surrounding water level (Figure 4d). Figure 4e compares the numerically calculated and experimentally measured multi‐fold meta‐display images, including the prior‐/post‐ immersion near‐field displays and holographic images. Similar to Figure 3b, under the illumination of a linear polarized laser beam at the wavelength of 590 nm, the near‐field display is dynamically transformed from a butterfly pattern in air to a crab pattern in water. More intriguingly, the holographic image projected onto the far‐field screen is simultaneously switched from an airplane pattern to a ship. The holographic images here are designed to exhibit off‐axis images to avoid any stray light overlapping and interference with zeroth‐order diffraction. Overall, the measured meta‐display images are in excellent agreement with the simulated images predicted by the modified SAA method. More dynamic animation for the quad‐fold switchable meta‐display can be found in Movie S2 (Supporting Information). Such water‐immersion switching approach features robust repeatability and large tuning area, without requiring any complicated local contacts or hard‐to‐implement stimuli. As an outlook, future meta‐display designs involving more amplitude levels and larger metasurface areas could create and encrypt more delicate and vivid‐coloring meta‐images.

## 3 Conclusion

In summary, we have proposed and experimentally demonstrated a facile water‐immersion tuning scheme to realize a multi‐field switchable meta‐display with independent‐encoding freedom. Such water‐immersion tuning scheme, for the first time, achieves the pixel‐programmable switch for both near‐ and far‐field meta‐display transformation, which goes beyond simple coloring alteration or on‐and‐off switching in previous attempts. Benefitting from large tuning area, great simplicity in operation and strong repeatability, we envision that the proposed water‐immersion approach for meta‐display will have potential applications in the next‐generation intelligent display technology with practical application in dynamic display/encryption, information anticounterfeit/storage, and optical sensors.

## 4 Experimental Section

### Sample Fabrication

First, a 2 nm thick Cr and consequently 70 nm thick Au film on the top of a fused silica substrate by thermal evaporation (evaporation rate of ≈0.9–1.1 Å s^−1^ and pressure of 1 × 10^−4^ Pa) was deposited. Afterward, the commercial HSQ preparation (SX AR‐N 8200) was diluted at 2:3 and rotated for 1 min at 4000 rpm to obtain a film thickness of ≈120 nm. After the prior‐baking at 150 °C hot plate for 10 min, the sample was then exposed and patterned by electron beam lithography (Raith eLINE Plus, 20 kV). For the post‐baking, the sample was heated for 10 min at 170 °C hot plate. Consequently, the sample was developed for 50 s and washed in water for 30 s. Finally, the 2 nm thick Cr and 30 nm thick Au were deposited by thermal evaporation without any lift‐off process.

### Numerical Simulation

The numerical simulations for the optical behaviors of the nanostructure screening are performed by the finite‐difference time‐domain (FDTD) method. The calculated domain of a meta‐pixel covering an area of 500 × 500 nm^2^ at the *x‐y* plane, is defined by the periodic boundaries along *x‐* and *y‐*directions and perfectly matched layers (PMLs) along *z‐*directions. Plane waves are launched normally incident to the meta‐pixel or pattern along the +*z‐*direction, and the spectra are collected by a field monitor placed behind the plane wave source. The surrounding environment is correspondingly changed depending on the immersion condition in air or water. The complex refractive index of Au is utilized from the data of Palik (0–2 µm), and the refractive indexes of HSQ and water are set to 1.461 and 1.33, respectively.

## Conflict of Interest

The authors declare no conflict of interest.

## Supporting information

Supporting Information

Supplemental Movie 1

Supplemental Movie 2

## Data Availability

The data that support the findings of this study are available from the corresponding author upon reasonable request.
