# Supplementary material for: Toward Water‐Immersion Programmable Meta‐Display
Source: Adv Sci (Weinh). 2022 Dec 18;10(5):2205581. doi: 10.1002/advs.202205581 (PMC9929123; doi:10.1002/advs.202205581)
Supplement: Supplementary file 1 — Supporting Information [file ADVS-10-2205581-s002.pdf]

ADVANCED SCIENCE
Open Access

Supporting Information

for *Adv. Sci.*, DOI 10.1002/advs.202205581

Toward Water-Immersion Programmable Meta-Display

Shuai Wan, Chenjie Dai, Zhe Li, Liangui Deng, Yangyang Shi, Wanlin Hu, Guoxing Zheng, Shuang Zhang and Zhongyang Li**

Supporting Information

Towards water-immersion programmable meta-display

Shuai Wan[1], Chenjie Dai[1], Zhe Li[1], Liangui Deng[1], Yangyang Shi[1], Wanlin Hu[1], Guoxing Zheng[1,2,3], Shuang Zhang[4], Zhongyang Li[1,2,3*]*

[1]Electronic Information School, Wuhan University, Wuhan 430072, China
[2]Wuhan Institute of Quantum Technology, Wuhan 430206, China
[3]School of Microelectronics, Wuhan University, Wuhan 430072, China
[4]Department of Physics, The University of Hong Kong, Pokfulam Road, Hong Kong 999077, China
*Corresponding author: Email: *shuzhang@hku.hk (S. Z.); zhongyangli@whu.edu.cn (Z. Y. L.)*

Section S1. Captured optical images of selected meta-pixels with multiple pattern sizes.

The optical images presented in **Figure S1** are seven selected meta-pixels with various geometrical parameters and pattern sizes at prior- and post-immersion states. The experimental viewing colors cover a large color gamut and become noticeably shifted between different immersion states. For different pattern sizes of 50 μm, 20 μm, 10 μm, 5 μm, and 2 μm, all can exhibit uniform and vivid coloring. The smallest meta-pixels is approximately 2 μm×2 μm, which indicates its capability to obtain a high-resolution display reaching ~ 12 700 dpi that is sufficient for advanced display applications. In addition, when designing meta-display devices, we have integrated several identical elements into one pixel (2×2 μm^2) to reduce the coupling effect between adjacent different elements.

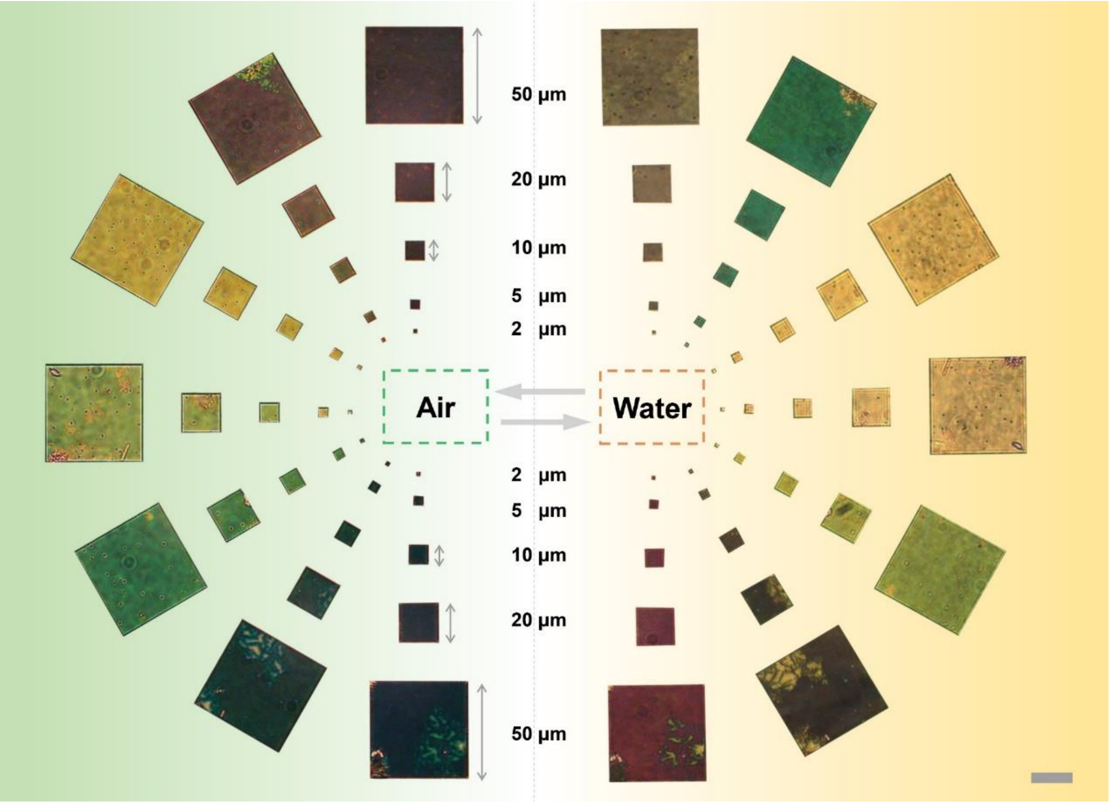

Figure S1. Optical images of the selected meta-pixels with different geometrical parameters and pattern sizes at prior- and post-immersion states. The pattern sizes vary from 2 μm to 50 μm. The scale bar is 10 μm.

Section S2. The switching time and the tunability of the water-immersion programmable meta-device.

The switching time for the water-immersion tuning scheme from the air state to water-immersion state is ~ 320 ms, and the switching time from water-immersion state to air state is ~ 30 ms. The complete switching cycle would be ~ 1350 ms (< 1.5 s) if the stable display time is 500 ms for each state.

The main factor impacting the switching time is controlling the fluid to immerse or remove from the pattern. In addition, the hydrophilicity and hydrophobicity of the structure and the viscosity of the selected liquid would also impact the modulation rate. We envision that our device in future work is possibly encapsulated into the commercialized microfluidic system, which would significantly increase and precisely control the modulation rate of the meta-display device. For instance, in the recent work (*Nat. Nanotechnol.* **2022**, *17*, 1097-1103), the combination of meta-optics devices and microfluidic systems was demonstrated to be an effective and practical strategy for tunable optical displays, and the modulation rate of the system is about 10 Hz (~ 100 ms).

Regarding the tunability of the platform, the pixel-level programmability relies on water-immersion tuning mechanism and changing the unit cells' geometric size. Therefore, the proposed platform enables dynamic switching between two specific programmed images rather than tuning to arbitrary images.

Regarding dynamically exhibiting arbitrary images, it requires the elaborate control of each unit, such as the previous works Nat. Commun. 2017, 8, 197 and Adv. Sci. 2020, 7, 1903382, demonstrated in the microwave region. However, it is difficult for the visible region with the similar method due to the much smaller pixel size. Here, our proposed platform develops the

water-immersion encoding capability to attempt dynamic switching between two specific programmable channels.

Section S3. Image quality analysis and the improvement potential of the water-immersion programmable meta-display.

Regarding the proposed design approach, the holographic images reconstructed by the modified simulated annealing algorithm (SAA) only take the spectral amplitude into account without considering the meta-pixel phase. As shown in **Figure S2**a, for the case of implementing the structural random phase into the far-field reconstruction, it shows that the image quality of the reconstructed holography hardly reduces with only slightly higher crosstalk between different display channels. Therefore, it is reasonable to approximate the structural random phase with the uniform phase to reduce the design complexity.

In the experimental verification, to reduce the design complexity and the written pattern size for electronic beam lithography, we select two amplitude levels with a pixel matrix size of 300×210 (Figure 4), which suggests its tremendous potential for achieving high-quality display applications. As for the further demonstration with more delicate display exhibition and switch, utilizing 24 amplitude levels with a larger pixel matrix size of 1080×720 could exhibit and encrypt higher image quality and more vivid meta-images, as shown in Figure S2b.

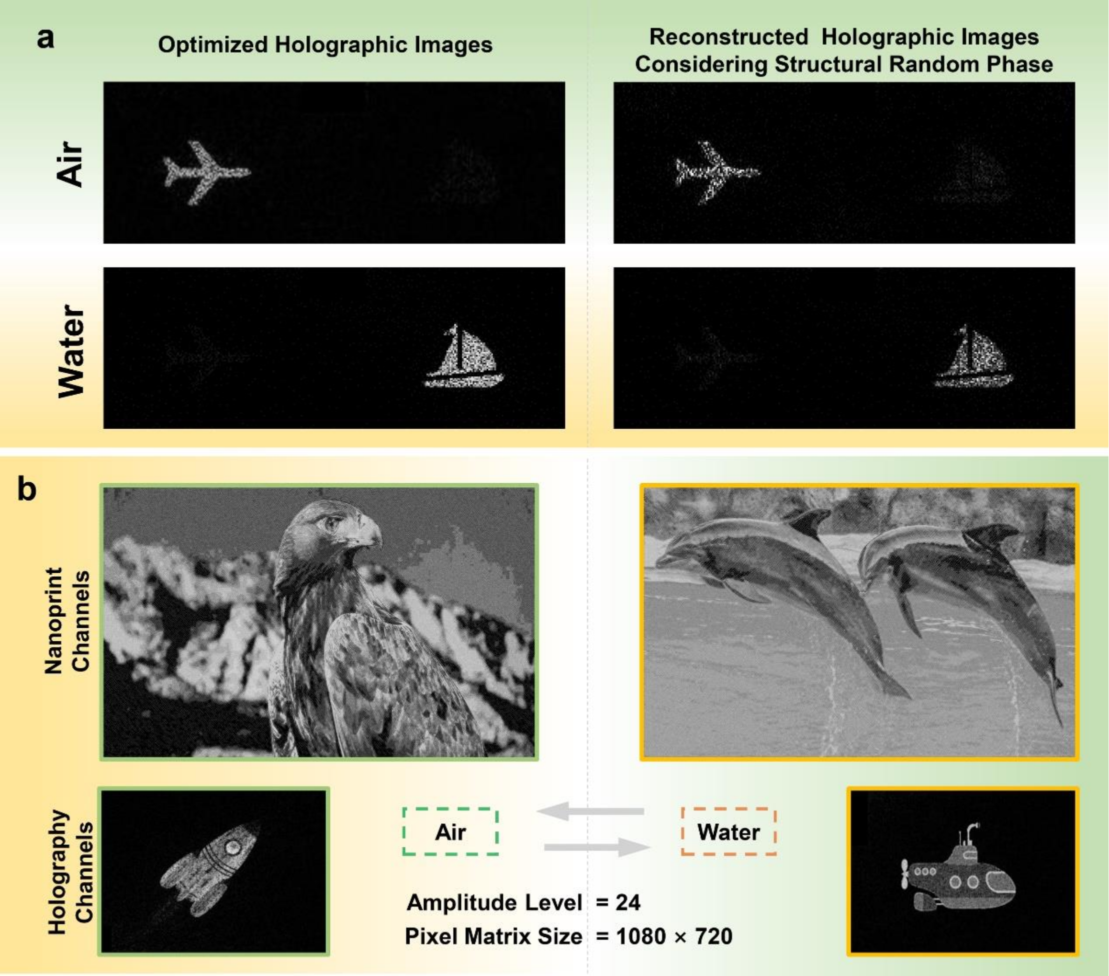

Figure S2. Image quality theoretical analysis and its improvement potential for the water-immersion programmable meta-display. a) Reconstruction comparison for holographic images with uniform phase and structural random phase of the metasurface array. b) Simulated independent-encode water-immersion quad-fold meta-display with 24 amplitude levels with a pixel matrix size of 1080×720. The reconstructed holographic images exhibit the respective designated area.

Movie S1. Dynamic switch and its strong repeatability for the nanoprint transformation animation during the water-immersion process.

Supplementary Movie S1 shows the experimental demonstration of repeatable dynamic switch for near-field nanoprint imaging, which provides additional visualization of the nanoprint

switch for Figure 3d. By immersing or drying the sample, the exhibited nanoprint image can be repeatably switched between a butterfly in air and a crab in water.

Movie S2. Dynamic switch for the multi-field meta-display transformation animation by tuning the immersion states.

Supplementary Movie S2 shows the experimental demonstration of dynamic **s**witch for the multi-field meta-imaging, which provides additional visualization of the near-field nanoprint and far-field holographic images switch for Figure 4e.

On one hand, by immersing or drying the sample, the exhibited nanoprint image can be dynamically switched between a butterfly in air and a crab in water. On the other hand, the dynamic holography transformation animation is successfully captured with a smartphone camera. The holographic images could be tuned to alternatively exhibit by dynamic controlling the immersion state of the sample in the capsule (Figure 4d).
